# Advances in epigenetic glioblastoma therapy

**DOI:** 10.18632/oncotarget.14612

**Published:** 2017-01-12

**Authors:** Dong Hoon Lee, Hyun-Wook Ryu, Hye-Rim Won, So Hee Kwon

**Affiliations:** ^1^ College of Pharmacy, Yonsei Institute of Pharmaceutical Sciences, Yonsei University, Incheon, Republic of Korea; ^2^ Department of Integrated OMICS for Biomedical Science, Yonsei University, Seoul, Republic of Korea

**Keywords:** histone deacetylase, histone deacetylase inhibitor, glioblastoma, epigenetic therapy

## Abstract

Glioblastoma multiforme (GBM) is the most lethal primary brain tumor in adults despite contemporary gold-standard first-line treatment strategies. This type of tumor recurs in virtually all patients and no commonly accepted standard treatment exists for the recurrent disease. Therefore, advances in all scientific and clinical aspects of GBM are urgently needed. Epigenetic mechanisms are one of the major factors contributing to the pathogenesis of cancers, including glioblastoma. Epigenetic modulators that regulate gene expression by altering the epigenome and non-histone proteins are being exploited as therapeutic drug targets. Over the last decade, numerous preclinical and clinical studies on histone deacetylase (HDAC) inhibitors have shown promising results in various cancers. This article provides an overview of the anticancer mechanisms of HDAC inhibitors and the role of HDAC isoforms in GBM. We also summarize current knowledge on HDAC inhibitors on the basis of preclinical studies and emerging clinical data.

## INTRODUCTION

Glioblastoma multiforme (GBM) is an aggressive, highly invasive, vascularized brain tumor [1]. Despite multimodal treatment, prognosis is unfortunately very poor; less than 5% of patients surviving at 5 years following initial diagnosis. Standard regimen includes maximum safe surgical resection followed by chemoradiation therapy [2]. Genetics, epigenetics, bacterial infection, and many other factors influence GBM oncogenesis, but the molecular mechanism underlying gliomagenesis is poorly understood [3, 4]. Conventional chemotherapy has limited efficacy in GBM due to poor blood-brain barrier (BBB) penetration, intratumor heterogeneity, intrinsic GBM resistance, and nonspecific toxicity [1, 5]. Based on successful preclinical studies, many clinical trials have tested the efficacy of novel therapies, but improved survival for patients with GBM has been limitedly achieved over the past few decades [6]. Therefore, further work is urgently required to discover novel therapeutic targets and develop more effective combination strategies for GBM treatment.

Histone deacetylase (HDAC) inhibitors have evoked great interest for the treatment of numerous malignancies because they are able to change transcriptomic profiles to promote tumor cell death. Hallmark features of GBM, including enhanced proliferation, invasion and migration, angiogenesis, and resistance to apoptosis, are targeted by HDAC inhibitors. The HDAC inhibitors vorinostat, panobinostat, valproic acid (VPA), and entinostat are well-studied epigenetic agents that effectively radiosensitize various tumors, including GBM [7]. HDAC inhibitor is among the successful examples of epigenetic therapy. Several HDAC inhibitors are US FDA approved, including the hydroxamic acid-based compounds vorinostat, panobinostat, belinostat, and the depsipeptide romidepsin for hematological malignancies [8]. Vorinostat [9–12] and VPA [13] are currently being tested in clinical trials on GBM as either monotherapies or combination therapies. The other FDA-approved epigenetic drugs, azacytidine and decitabine, DNA methyltransferase inhibitors [14], have not been clinically tested to evaluate its anticancer effect on GBM. Although drugs targeting histone methyltransferases and demethylases have considerable potential, their specific effects and the stability of such effect must be elucidated in greater detail to develop as antitumor agents. [15]. To the best of our knowledge, no drugs that target histone methylation or epigenetic readers are FDA approved or under clinical trials. To date, of the epigenetic agents, only HDAC inhibitors have been investigated in clinical trials as antitumor agents against GBM. Thus, this review focuses on recent studies that highlight the role of HDAC isoforms and discuss the preclinical and clinical data on HDAC inhibitors as therapeutic agents for GBM.

## GLIOBLASTOMA MULTIFORME

Glioblastoma is the most common malignant primary tumor of the central nervous system in adults. GBM represents approximately half of all gliomas and 15% of primary brain tumors [1]. WHO grade IV GBM, which is the highest grade glioma, is divided into two major classes: “primary” and “secondary.” A vast majority of GBMs arise *de novo* as primary GBMs in elderly patients. Secondary GBMs, those that arise from a pre-existing glioma of WHO grade II or III, are less frequent [16, 17]. Most secondary GBMs develop in younger patients ( < 45 years). The disease incidence continues to increase with age and the median age at diagnosis is 64 years. Survival rates are poor; only approximately 34% of patients survived for 1 year, 12% for 2 years, and less than 5% for 5 years from the time of diagnosis. Older age and incomplete surgical resection are associated with poor survival [18, 19]. GBM remains one of the deadliest of malignancies, with limited treatment options and a high rate of recurrence [2, 20, 21].

While histologically identified ischemic necrosis and elevated microvascular proliferation, GBM is more accurately characterized and distinguished by its genomic and epigenomic profiles [19]. The Cancer Genome Atlas (TCGA) created a gene expression-based molecular classification system in which GBM is categorized into mesenchymal, classical, neural, and proneural subtypes [22]. These subtypes were compared with the corresponding normal neural cell types to determine the possible cellular origin for each of these tumors; correlations between subtype and clinical response were determined. TCGA research network reported that three signaling pathways are frequently modified in GBM: receptor tyrosine kinase (RTK)/Ras/phosphoinositide 3-kinase (PI3K), p53, and retinoblastoma (Rb) signaling. In adults, components of the RTK/Ras/PI3K pathway are mutated in 88% of GBMs, those of the p53 pathway in 87%, and those of the Rb pathway in 78%. Mutations such as amplification of the epidermal growth factor receptor (EGFR) can be found in 45% of GBMs, gain of PI3K function in 15%, and loss of phosphatase and tensin homolog (PTEN) in 36% [23, 24]. These discoveries have led to a better understanding of the molecular signature of GBM and have revealed numerous consistent changes in genes and pathways [4, 16, 22, 25, 26]. However, there is still an unmet need to translate these findings into clinical practice, identify predictive biomarkers, and improve outcomes for patients with GBM.

## CURRENT STANDARD TREATMENT

The current first-line standard regimen for GBM is an aggressive combination therapy, including maximum safe surgical resection and adjuvant radiotherapy with concurrent and adjuvant temozolomide chemotherapy [2, 16]. Surgical resection is often compromised by the diffusely infiltrative nature of gliomas, which recur even after gross total resection. In addition, these tumors often invade critical neurological structures, precluding complete surgical resection [19, 27]. Radiation therapy following surgery increases the median survival times ranged from 14 to 36 weeks [28]. The benefits of treatment with radiation were initially established using whole brain radiotherapy, but improved technology (e.g., field radiation therapy) has markedly reduced the associated side effects [19]. A total radiation dose of 60 Gy delivered to the tumor provides the maximum survival benefit [29].

The addition of the alkylating agent temozolomide to postoperative radiation or concurrent temozolomide and radiotherapy is the only chemotherapeutic regimen that significantly improves the overall survival (OS) of patients with GBM. The methylation status of MGMT (O^6^-methylguanine-DNA methyltransferase), a DNA-repair gene, is used as a GBM predictor because it is the major relevant biomarker for the response to temozolomide treatment [24]. Silencing of MGMT expression by promoter methylation impairs the ability of tumor cells to repair the DNA damage induced by temozolomide, further decreasing tumor cell survival [30]. Patients whose tumors have the unmethylated MGMT gene promoter also experience a modest but less significant benefit from the addition of temozolomide. Thus, combined treatment with temozolomide and radiation remains the standard regimen for all patients with GBM [19, 31]. However, the improved 2-year survival with temozolomide treatment is only in 27% [24], which is still unsatisfactory.

Currently, bevacizumab (Avastin) is the only approved therapeutic agent for the treatment of patients with recurrent GBM. Bevacizumab is a humanized therapeutic antibody that specifically binds to vascular endothelial growth factor (VEGF)-A, disrupting VEGF-VEGF receptor interaction and preventing angiogenesis [32, 33]. Because GBM tumors are particularly vascular and overexpress numerous angiogenic factors, antiangiogenic therapy is efficient. A phase II trial of combined treatment with bevacizumab and irinotecan (a topoisomerase 1 inhibitor) in recurrent GBM showed increased OS from 4.1 to 9.2 months [11]. The 6-month and 12-month survival rates were 77% and 31%, respectively [33–35]. However, patients who had received previous bevacizumab therapy had shorted PFS and OS. Bevacizumab was subsequently investigated in phase III trials for newly diagnosed GBM, but there was no effect on overall patient survival. In addition, phase III trials are currently being tested to evaluate the efficacy of bevacizumab with temozolomide and radiotherapy for newly diagnosed GBM (NCT00884741 and NCT00943826) [19, 36].

## HDAC EXPRESSION IN GBM

The human genome contains 18 known HDACs, which are grouped into four classes on basis of phylogenetic analysis [37, 38]: class I (HDAC1, 2, 3 and 8), IIa (HDAC4, 5, 7, 9) and IIb (HDAC6, 10), III (SIRT1-7) and VI (HDAC11). The HDAC family is separated into Zn^2+^-dependent (classes I, II and IV) and Zn^2+^-independent (class III), nicotinamide-adenine dinucleotide-dependent enzymes. Class I, II and IV HDACs are also referred to as classical HDACs. Most HDAC inhibitors available as anticancer agents target class I and II HDACs. Class I HDACs are primarily nuclear proteins and have histones as principle target substrates. Class I HDACs are ubiquitously expressed in all tissues whereas class II HDACs are tissue-specifically expressed [39]. Class II HDACs shuttle between the nucleus and cytoplasm and have histone and non-histone proteins as primary targets. HDAC11 (class IV) is phylogenetically most closely related to HDAC3 and 8 but also has some features of class II HDACs [40]. Class III HDAC is also called sirtuins (SIRT) and comprises seven SIRT isoforms (SIRT1-7), which differ in their substrate specificities, functions and subcellular localization [41].

HDACs are overexpressed and mutated in various solid and hematologic malignancies and play key roles in tumorigenesis [38, 39]. The expression of individual HDACs is largely inversely correlated with disease-free and overall survival rates. The aberrant expression of HDACs correlates with a poor prognosis [42]. However, the expression and functions of HDACs in GBM are not well characterized. Recent studies have begun to focus on the expression patterns of HDACs in GBM. GBM cells and primary GBM tissues exhibit slightly and variably increased HDAC1, 3 and 6 expression levels compared to non-neoplastic brain tissues at both the mRNA and protein levels [43]. This result was further confirmed in silico using the REMBRANDT glioma dataset available through a GlioVis online application. In particular, HDAC1 and 3 expression levels correlate with WHO tumor grades, with the highest expression levels occurring in GBM samples. Furthermore, Kaplan-Meier survival curve analyses show that HDAC3 expression is associated with a poor survival of in GBM patients. Another study demonstrates that HDAC9 is overexpressed in prognostically poor GBM patients using TCGA and French's datasets [44]. This result was further confirmed in primary GBM tissues and cell lines. Class III HDAC SIRT2 positively correlates with GBM malignant progression and inversely correlated with the survival time of patients with GBM [45]. In contrast, SIRT1 and 6 are downregulated in GBM tissues and cell lines [46–48]. The role of SIRT in GBM is currently under debate due to conflicting findings suggesting that SIRT acts as a tumor suppressor or as an oncogene [49, 50]. SIRT inhibitors have not been clinically tested to evaluate their anticancer effect on GBM. Thus, this review focuses on Zn^2+^-dependent HDACs and their inhibitors.

## HDAC INHIBITORS AS CANCER THERAPY FOR GBM

**Figure 1:**
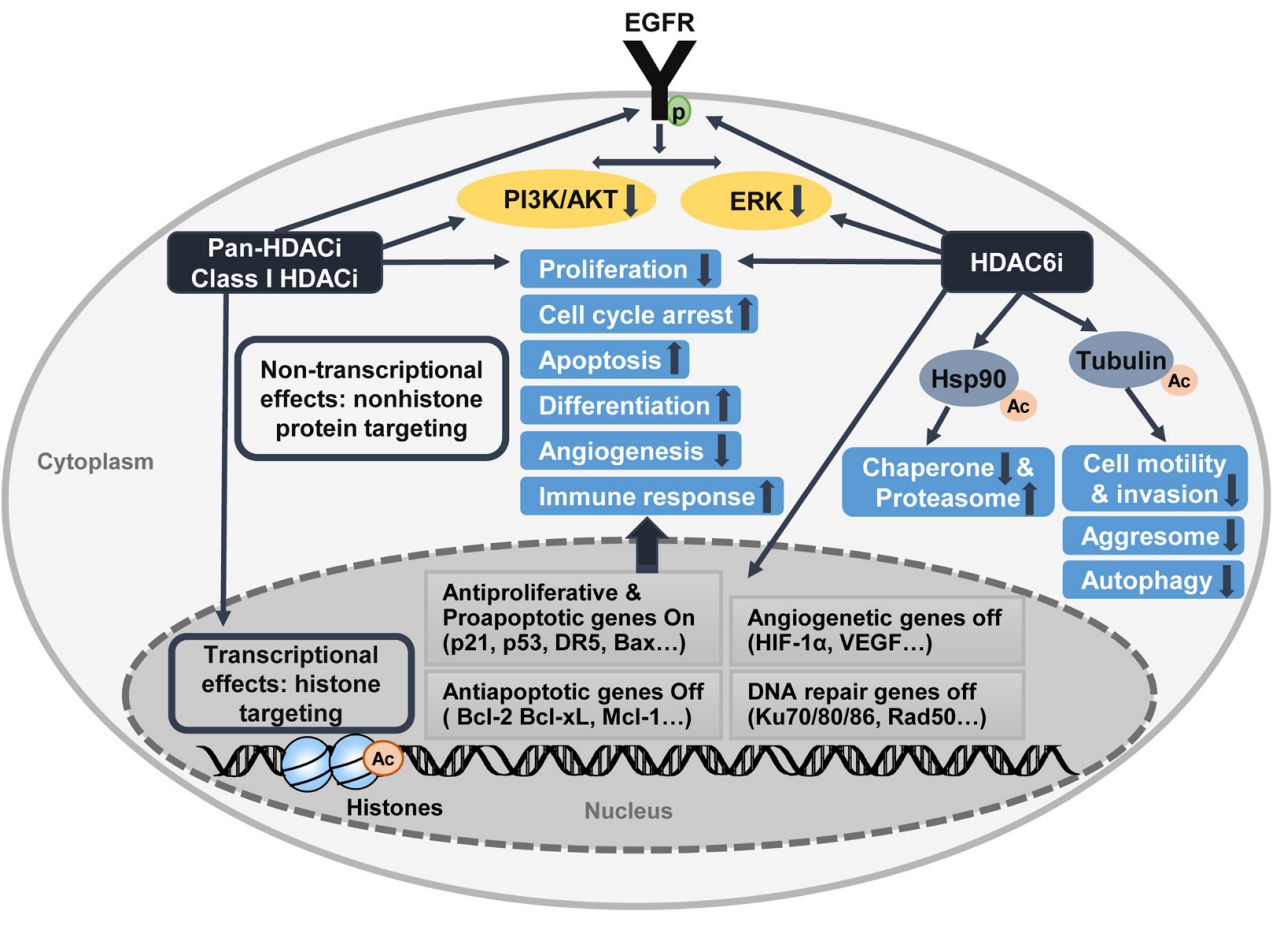
Antitumoral activity of HDAC inhibitors

HDAC inhibitors are classified as epigenetic agents that target the aberrant epigenetic characteristics of tumor cells. Epigenetic alterations modulate cellular phenotype through changes in gene expression without modifying the DNA sequence [19]. HDAC inhibitors are known as effective therapeutic anticancer agents *via* multiple mechanisms, including the induction of cell-cycle arrest, differentiation, senescence, intrinsic and extrinsic apoptosis, mitotic cell death, autophagic cell death, generation of reactive oxygen species, inhibition of angiogenesis and metastasis, and improvement in tumor immunity [8, 51] (Table 1 and Figure 1). Because these diverse effects on cancer cells overlap, HDAC inhibitors are very attractive as single agents and in combination with other therapies (Table 2). HDAC inhibitors are a promising class of therapeutic agents that are under investigation for treating different types of tumors, including GBM.

**Table 1:** Antitumor activity of HDAC inhibitors

Biological effects	Key effects of HDAC inhibitors
∙ *Direct effects on tumor cells*	
Cell death	∙ Induction of apoptosis through the intrinsic and extrinsic apoptosis pathways ∙ Enhanced ROS production and decreased production of free radical scavengers ∙ Immunogenic cell death
DNA damage and repair	∙ Accumulation of DNA damage through transcriptional downregulation or impaired function of DNA repair proteins
Cell cycle arrest	∙ Induction of cell cycle arrest
Senescence	∙ Induction of senescence
Autophagy	∙ Induction of autophagy
Differentiation	∙ Induction of differentiation
Tumor immunogenicity	∙ Enhanced immunogenicity ∙ Enhanced antigenicity of tumor cells
∙ *Indirect effects on tumor cells*	
Immunomodulation	∙ Inhibition of dendritic cell differentiation and function ∙ Cytotoxicity to macrophages, neutrophils, and eosinophils ∙ Induction of apoptosis in proliferating B cells ∙ Increased tumor killing by NK cells and cytotoxic T cells ∙ Increased differentiation and function of CD8^+^ T cells ∙ Inhibition immunosuppressive functions of regulatory T cells ∙ Suppression of inflammatory cytokine production ∙ Increased expression of PD-L1
Inhibition of angiogenesis	∙ Suppressed expression of pro-angiogenic genes
Inhibition of metastasis	∙ Suppressed expression of pro-metastatic genes ∙ Increased expression of anti-metastatic genes
Glucose metabolism	∙ Inhibition of glucose utilization

Abbreviations: NK, natural killer; ROS, reactive oxygen species; PD-L1, programmed death-ligand 1.

**Table 2:** Preclinical studies on HDAC inhibitors as therapeutic agents for GBM

HDAC inhibitor	Chemotherapeutic or biological agents	Radiation therapy (RT)	Function	Ref
**Valproic acid**	-	RT	Protection of normal hippocampal neurons Radiosensitizer up to 12 h after post-irradiation	[67]
**Vorinostat** **(SAHA)**	-	RT	Induction of chromatin decondensation Increased DNA DSBs Induction of apoptosis	[65,66]
	Bcl2 inhibitor (obatoclax)	RT	Synergistic apoptotic GBM cell death Overcome resistance to SAHA as a radiosensitizer	[66]
	KDM1A inhibitor (tranylcypromine)	-	Synergistic apoptotic GBM cell death	[68]
	PARP inhibitor (olaparib)	-	Decline of DDR marker expressions Impaired cell cycle progression Synergistic apoptotic GBM cell death	[6]
**Panobinostat** **(LBH589)**	-	RT	Induction of chromatin decondensation Increased DNA DSBs Induction of apoptosis	[64, 66]
	Bcl2 inhibitor (obatoclax)	RT	Synergistic apoptotic GBM cell death Overcome resistance to LBH589 as a radiosensitizer	[66]
**Entinostat** **(MS-275)**	-	RT	Minimal radiosensitizer after post-irradiation	[53]
**HDAC6 inhibitors** **Ricolinostat** **(ACY-1215)**, **Tutastatin A**, **CAY10603**	Temozolomide	-	Inactivation of the EGFR pathway Inhibition of cell proliferation Induction of apoptosis Impaired spheroid formation Overcome resistance to TMZ	[80]

## PRECLINICAL STUDIES OF HDACS AND HDAC INHIBITORS IN GBM

### Pan-Histone deacetylase inhibitors as radiosensitizers

Several preclinical studies have revealed that HDAC inhibitors act as potent radiosensitizers in various cancers, including GBM [52–55], breast cancer [56], colorectal cancer [57], head and neck cancer [58], non-small-cell lung cancer [59], melanoma [60], and prostate cancer [53]. The exact molecular mechanism underlying HDAC inhibitor-induced radiosensitization remains elusive. However, evidence suggests that it partially involves the inhibition of the DNA damage repair response [19]. HDAC inhibitors block DNA double-strand break (DSB) repair following radiation, as evidenced by the continuous expression of phosphorylated H2AX (γH2AX) Several HDAC inhibitors delay the dispersal of γH2AX foci in irradiated cells [52, 53, 55, 57, 59, 60]. Despite the undefined mechanism for this defective DNA DSB repair process, HDAC inhibitors may affect at least two components of this repair process. They induce the downregulation of DNA repair proteins, including Ku70, Ku80, Ku86, Rad50, and Rad51 [59, 62]. Also, the binding of HDACs to DNA damage response proteins may play a key role in HDAC inhibitor-induced radiosensitization [19, 63].

Furthermore, HDAC inhibitors may influence the response of tumor cells to radiation by changing the chromatin structure. Pan-HDAC inhibitor LBH589 (panobinostat) treatment induced chromatin relaxation and this chromatin decondensation correlated with increased levels of DNA DSBs and radiosensitivity [19, 64]. Thus, HDAC inhibitor-induced chromatin decompaction may increase DNA DSBs induced by radiation, ultimately increasing tumor cell death. Another pan-HDAC inhibitor, suberoylanilide hydroxamic acid (SAHA; vorinostat), has shown a similar effect in GBM [65]. DNA damage response markers and antiapoptotic proteins predict the radiosensitization efficacy of vorinostat and panobinostat in patient-derived GBM cells [66]. Responses to SAHA and LBH589 correlate with pChk2 and Bcl-xL levels. In patient-derived GBM stem-like cells, the Bcl-2 inhibitor obatoclax is reported to abrogate resistance to SAHA and LBH589 as radiosensitizers [66]. Other class I HDAC inhibitors (MS-275 and VPA) were also tested for administration after irradiation, with differing results. MS-275 displayed only minimal radiosensitization after irradiation [19, 53]. In contrast, VPA effectively radiosensitized cells when administered up to 24 h after radiation treatment, although the augmented degree was not strong compared with that of cells treated both before and after irradiation [19, 67]. Therefore, these data indicate that sufficiently high HDAC inhibitor levels should be maintained in tumor cells both before and after irradiation because the cells seek to repair the DNA damage induced by radiation [19, 29]. Overall, HDAC inhibitors seem to prevent DNA DSB repair, resulting in increased tumor cell death.

#### Pan-HDAC inhibitors as combination drugs

In addition to radiosensitizers, HDAC inhibitors have been used as chemosensitizers in GBM [68, 69]. SAHA affects gene expression patterns and proliferation of glioma cells. After SAHA treatment in GBM cell lines, the expression level of many proapoptotic, antiproliferative genes (*DR5, TNFα, p21*, and *p27*) increased and that of many antiapoptotic, progrowth genes (*CDK2, CDK4*, and the genes encoding cyclins D1 and D2) decreased [70]. HDAC1/2 and histone H3K4 demethylase (LSD1/KDM1) are components of common nuclear corepressor complexes and the acetylation status of adjacent histone residues affects the activity of LSD1 [71]. These results provide the rationale for using dual inhibitors of LSD1 and HDAC for cancer treatment. The inhibition of LSD1 renders GBM cells sensitive to SAHA [68]. Combined treatment with tranylcypromine and SAHA synergistically induces apoptotic cell death in GBM cells. These data suggest that LSD1 and HDACs cooperatively modulate the key pathways of GBM cell death and verify the combined administration of LSD1 and HDAC inhibitors as a therapeutic strategy for GBM.

Several studies report constitutively active DNA damage response in malignant gliomas caused by continuous oxidative and replicative stress [6, 72–74]. In addition to constitutive activation of DNA damage response, genomic instability causes therapeutic resistance and high recurrence rates. Inhibition of poly (ADP-ribose) polymerase (PARP) efficiently eradicates GBM cells, either alone or in combination with chemoradiation [74, 75]. PARP inhibition increases the radiosensitivity of radioresistant GBM cells. Currently, a phase I trial of olaparib (AZD2281; a potent inhibitor of PARP1/2) in conjunction with temozolomide is being investigated in patients with relapsed GBM (ClinicalTrials.gov ID: NCT01390571) [6]. The expression of all DNA damage response markers (BRCA1, Rad51, and PARP1) was observed to further decrease when combined with SAHA and olaparib. This combination treatment synergistically reduced GBM cell survival, induced apoptosis, and inhibited cell-cycle progression [6]. These data also provide a preclinical rationale for combined treatment with SAHA and olaparib, which are already under investigation individually in clinical trials.

### Isoform-selective HDAC inhibitors

Most of the GBM studies to date have focused on testing the antitumor effects of pan-HDAC inhibitors such as vorinostat and VPA rather than evaluating the role of HDAC in GBM. Despite some encouraging results from preclinical studies, early clinical trials showed only modest therapeutic benefits. Therefore, the value of pan-HDAC inhibitors in clinical practice is needed for further verification in larger prospective trials to address the function of each HDAC isoform in GBM. Few recent studies investigated the role of HDAC isoforms in GBM. New molecules that target individual HDACs are under preclinical development (such as PCI-34051, which targets HDAC8) or clinical trials (such as ACY-1215, which targets HDAC6).

#### HDAC6

HDAC6 belongs to class IIb HDAC family. This enzyme deacetylates various substrates, including cortactin, Hsp90, and α-tubulin in the cytoplasm and nucleus [37]. HDAC6 controls both epigenetic and non-epigenetic mechanisms by shuttling between these two cellular compartments. An increasing number of studies suggest that HDAC6 is also a pivotal regulator of cancer-related signaling pathways, including the EGFR, mitogen-activated protein kinase (MAPK), protein kinase B, and p53 signaling pathways. These findings indicate HDAC6 as a potential therapeutic target for cancer therapy [76]. Aberrant expression patterns of HDAC6 are found in various cancers, including breast cancer [77], oral squamous cell carcinoma [78], ovarian cancer [79], GBM [80], and mouse tumor models. Recently, Wang et al. reported that HDAC6 increases proliferation and imparts temozolomide resistance in GBM [80]. HDAC6 is overexpressed in GBM tissues and cell lines. HDAC6 overexpression facilitates the proliferation and spheroid formation of GBM cells and renders GBM cells resistant to temozolomide. Conversely, knockdown or inactivation of HDAC6 prevents cell proliferation, induces apoptosis, hinders spheroid formation, and renders GBM cells more sensitive to temozolomide. Moreover, temozolomide resistance is associated with activation of EGFR and increased expression of HDAC6. The HDAC6 inhibitors (ACY-1215, tubastatin A, and CAY10603) abrogate temozolomide resistance by decreasing and inactivating EGFR protein. These data imply that the inhibition of HDAC6 is a novel approach for treating GBM and overcoming resistance to temozolomide. ACY-1215 (ricolinostat), a leading HDAC6-selecitve inhibitor, is currently being tested in advanced clinical trials for hematological malignancies (myeloma and lymphoid malignancies) [81, 82]. Thus, these studies and the fact that ACY-1215 is already under clinical trials imply that HDAC6 inhibitors are worthy of consideration for further clinical tiral in GBM patients.

#### HDAC9

HDAC9 is a member of the class IIa HDAC family and controls regulatory T cell function, cardiac growth, and muscle differentiation [83–85]. It has been reported that HDAC9 expression is significantly upregulated in cervical cancer [86], medulloblastoma [87], acute lymphoblastic leukemia [88], and GBM [44]. HDAC9 is overexpressed in GBM patients who have a poor prognosis. HDAC9 promotes GBM proliferation and tumor formation *via* activation of the transcription coactivator with PDZ-binding motif (TAZ)-mediated EGFR pathway [44]. HDAC9 directly interacts with TAZ, an oncogene and an essential downstream effector of the Hippo pathway. Depletion of HDAC9 reduces the expression of TAZ. A significant effort is underway to find new molecules targeting class IIa HDACs, including HDAC9. However, to date, no HDAC9-specific inhibitors are available. Nevertheless, these results provide new evidence of a promising target for GBM treatment.

#### HDAC2

HDAC2 is a member of the class I HDAC family. High expression of HDAC2 has been reported in GBM cells [89]. Depletion of HDAC2 by siRNA suppresses proliferation, migration, and invasion of GBM cells and renders the cells sensitive to temozolomide. HDAC2 depletion significantly downregulates the mRNA and protein expression of MRP1 with no effect on ABCB1 and ABCG2. Schisandrin B, a specific inhibitor of MRP1, further enhances the temozolomide sensitivity in HDAC2 knockdown GBM cells. This finding suggests that HDAC2 is a viable target for GBM therapy and improves the efficiency of temozolomide therapy. However, to date, no HDAC2-specific inhibitors are available.

## CLINICAL TRIALS OF HDAC INHIBITORS IN GBM

Vorinostat, depsipeptide, panobinostat, and belinostat are the FDA-approved HDAC inhibitors for cancer therapy; these drugs are used specifically for the treatment of refractory cutaneous T-cell lymphoma (CTCL), peripheral T-cell lymphoma (PTCL), and multiple myeloma [8]. Numerous clinical trials are evaluating the safety and efficacy of other HDAC inhibitors, used singly or in combination, for the treatment of various malignancies [19] (https://clinicaltrials.gov). In general, the side effects of HDAC inhibitors include dehydration, diarrhea, fatigue, nausea, thrombocytopenia, lymphopenia, neutropenia, and prolonged QT [19, 90]. Despite favorable toxicity profiles and reversible adverse effects, HDAC inhibitors seem to be not sufficient as monotherapies in solid tumors compared with current standard cancer therapies, partly because of their poor pharmacokinetic properties [11, 91]. However, the potential of HDAC inhibitors as cancer therapeutic agents is apparent from clinical trials combining HDAC inhibitors with chemotherapies or targeted therapies. Table 3 summarizes the ongoing clinical trials of HDAC inhibitors in GBM.

**Table 3:** Current clinical trials on HDAC inhibitors in GBM

HDAC inhibitor	Chemotherapeutic or biological agents		Radiation therapy (RT)	Type of malignancy	Phase	Trial identifier
**Valproic acid**	Temozolomide		RT	GBM that has not been previously treated with chemotherapy or radiation	2	NCT00302159
**Vorinostat**	-		-	Progressive or recurrent glioblastoma	2	NCT00238303
	Temozolomide		-	Malignant glioma: glioblastoma	1	NCT00268385
	Temozolomide		RT	Newly diagnosed glioblastoma	1,2	NCT00731731
	Temozolomide + isotretinoin		-	Recurrent glioblastoma	1,2	NCT00555399
	Bortezomib		-	Progressive, recurrent glioblastoma	2	NCT00641706
	Bevacizumab		-	Recurrent glioblastoma	2	NCT01738646
	Temozolomide + bevacizumab		-	Recurrent glioblastoma	1,2	NCT00939991
	Bevacizumab + irinotecan		-	Recurrent glioblastoma	1	NCT00762255
**Belinostat**	Temozolomide		RT	Newly diagnosed glioblastoma	2	NCT02137759
**Romidepsin**		-	-	Recurrent high grade gliomas: glioblastoma	1,2	NCT00085540

### Vorinostat

Vorinostat is a small-molecule inhibitor of human class I and II HDACs that was the first FDA-approved HDAC inhibitor for the treatment of refractory CTCL. It has been reported that vorinostat can penetrate BBB and possesses antitumor effects in glioma models [70, 92]. Vorinostat is the most advanced HDAC inhibitor to enter clinical trials in GBM and is well tolerated as a monotherapy as well as combination therapy in recurrent GBM. A phase II trial tested the efficacy of vorinostat in patients with recurrent GBM [11, 33]. A total complete response (CR) or partial response (PR) occurred in only 3% of patients. Median progression-free survival (PFS) was 1.9 months and 6-month PFS was 17%. This trial showed modest monotherapy activity of vorinostat with a median OS of 5.7 months in recurrent GBM. There are multiple ongoing phase II trials of vorinostat in conjunction with targeted agents, temozolomide, and radiotherapy. A phase I trial of vorinostat in conjunction with temozolomide was well tolerated in patients with high-grade glioma, although thrombocytopenia and a related grade V hemorrhage were dose-limiting toxicities (ClinicalTrials.gov ID: NCT00268385). A phase I/II trial of vorinostat with radiotherapy and concomitant temozolomide demonstrated reasonable tolerability in newly diagnosed GBM, although the phase II efficacy information is not yet published (NCT00731731). A phase I/II trial of vorinostat, temozolomide, and isotretinoin in recurrent GBM is underway (NCT00555399). Another phase II trial investigated the effects of combination treatment of vorinostat with bortezomib (a proteasome inhibitor) in recurrent GBM [10, 33]. However, the trial was stopped because patients did not obtain 6-month PFS on interim analysis. The reduction of antitumor activity of bortezomib in GBM is likely due to lack of penetration of the BBB [93]. A phase I trial of vorinostat in combination with bevacizumab and irinotecan (a topoisomerase I inhibitor) in recurrent GBM found the same maximum tolerated dose for vorinostat with less thrombocytopenia. In addition, this study showed improved PFS and OS compared to that of vorinostat alone (NCT00762255) [9]. A phase II trial of vorinostat and bevacizumab for recurrent GBM is ongoing (NCT01738646).

### Panobinostat

Panobinostat (LBH589) is a potent, small-molecule inhibitor of class I, II, and IV HDACs that was FDA-approved for the treatment of multiple myeloma [94]. Panobinostat shows antitumor and antiangiogenic effects in glioma. A phase II trial of panobinostat in combination with bevacizumab in recurrent GBM was well tolerated. However, the trial was terminated because combination regimen did not significantly improve PFS at 6 month compared to historical controls of bevacizumab monotherapy (NCT00859222) [95].

### Valproic acid

VPA is a class I HDAC inhibitor as well as an antiepileptic drug [19], has a low toxicity profile [96] and effectively crosses BBB [97]. VPA showed impressive preclinical efficacy as a radiosensitizer in glioma cells at a dose comparable to that achievable clinically [98–101]. In contrast, it had a radioprotective effect on normal brain tissue and hippocampal neurons [102, 103]. Several retrospective studies analyzed the effects of VPA on the survival of GBM patients [104–106]. Although these results have suggested favorable effects of VPA, whether VPA improves the OS of GBM patients is debatable [107]. However, a phase II trial of VPA, temozolomide, and concurrent radiotherapy for GBM patients was investigated and promising results were recently reported [13]. The median OS is reported to be 29.6 months in patients with newly diagnosed GBM. The most common grade III/IV toxicities of the combination regimen are metabolic and laboratory toxicities (8%), neurological toxicity (11%), and blood and bone marrow toxicity (32%) (NCT00302159) [107], which seem to be well tolerated. Based on the considerable preclinical and retrospective data, VPA is considered to be one of the most promising agents for GBM treatment, but prospective data are still limited [107]. Further investigations are needed to assess its efficacy and clarify the optimal treatment.

### Romidepsin

Romidepsin (FK228) is a class I HDAC inhibitor [108] and was the second FDA-approved HDAC inhibitor for the treatment of refractory CTCL and PTCL [109]. It induces apoptosis and inhibits proliferation and metastasis of GBM cells [110]. Romidepsin was studied in a phase I/II trial on patients with recurrent high-grade gliomas, but at the standard dose and schedule, it was ineffective for patients with recurrent GBM (NCT00085540) [111].

## CONCLUSIONs

Despite advances in therapeutics and diagnostics, the prognosis of GBM is still poor, and clinically relevant biomarkers have not been established. Due to the heterogeneity of GBM tumors, new strategies have shown clinical limits in terms of efficacy and side effects. We need to understand the complexity of GBM to offer insight into the prognosis and management of this incurable disease. GBM tumorigenesis and chemoresistance are mediated by multiple factors, suggesting that multitargeted strategies are more efficient. Therefore, classification of GMB patients based on genetic, epigenetic, and transcriptional profiling data might be beneficial for selecting drugs for their treatment and predicting patient outcomes. Indeed, progress in the molecular classification of GBM contributes to develop more effective targeted therapeutic agents and combination strategies and to predict patient outcome. However, this progress is still unsatisfactory. We should pursue new discoveries that come from basic science and translate these scientific findings into effective clinical practice.

## Abbreviations

BBB: blood-brain barrier
CR: complete response
CTCL: cutaneous T-cell lymphoma
DSB: DNA double-strand break
EGFR: epidermal growth factor receptor
GBM: glioblastoma multiforme
HDAC: histone deacetylase
LSD1/KDM1A: lysine-specific histone demethylase 1A
MAPK: mitogen-activated protein kinase
MGMT: O^6^-methylguanine-DNA methyltransferase
OS: overall survival
PARP: poly(ADP-ribose) polymerase
Rb: retinoblastoma
PFS: progression-free survival
PKB/Akt: protein kinase B
PR: partial response
PTCL: peripheral T-cell lymphoma
RTK: receptor tyrosine kinase
SAHA: suberoylanilide hydroxamic acid
Sirtuin: SIRT
TCGA: The Cancer Genome Atlas
VPA: valproic acid
VEGF: vascular endothelial growth factor

## References

[1] Ostrom QT, Gittleman H, Liao P, Rouse C, Chen Y, Dowling J, Wolinsky Y, Kruchko C, Barnholtz-Sloan J (2014). CBTRUS statistical report: primary brain and central nervous system tumors diagnosed in the United States in 2007-2011. Neuro Oncol.

[2] Stupp R, Mason WP, van den Bent MJ, Weller M, Fisher B, Taphoorn MJ, Belanger K, Brandes AA, Marosi C, Bogdahn U, Curschmann J, Janzer RC, Ludwin SK, Gorlia T, Allgeier A, Lacombe D (2005). Radiotherapy plus concomitant and adjuvant temozolomide for glioblastoma. N Engl J Med.

[3] Network TC (2013). Corrigendum: Comprehensive genomic characterization defines human glioblastoma genes and core pathways. Nature.

[4] Cancer Genome Atlas Research N (2008). Comprehensive genomic characterization defines human glioblastoma genes and core pathways. Nature.

[5] Ellis HP, Greenslade M, Powell B, Spiteri I, Sottoriva A, Kurian KM (2015). Current challenges in glioblastoma: intratumour heterogeneity, residual disease, and models to predict disease recurrence. Front Oncol.

[6] Rasmussen RD, Gajjar MK, Jensen KE, Hamerlik P (2016). Enhanced efficacy of combined HDAC and PARP targeting in glioblastoma. Mol Oncol.

[7] Chinnaiyan P, Vallabhaneni G, Armstrong E, Huang SM, Harari PM (2005). Modulation of radiation response by histone deacetylase inhibition. Int J Radiat Oncol Biol Phys.

[8] Falkenberg KJ, Johnstone RW (2014). Histone deacetylases and their inhibitors in cancer, neurological diseases and immune disorders. Nat Rev Drug Discov.

[9] Chinnaiyan P, Chowdhary S, Potthast L, Prabhu A, Tsai YY, Sarcar B, Kahali S, Brem S, Yu HM, Rojiani A, Murtagh R, Pan E (2012). Phase I trial of vorinostat combined with bevacizumab and CPT-11 in recurrent glioblastoma. Neuro Oncol.

[10] Friday BB, Anderson SK, Buckner J, Yu C, Giannini C, Geoffroy F, Schwerkoske J, Mazurczak M, Gross H, Pajon E, Jaeckle K, Galanis E (2012). Phase II trial of vorinostat in combination with bortezomib in recurrent glioblastoma: a north central cancer treatment group study. Neuro Oncol.

[11] Galanis E, Jaeckle KA, Maurer MJ, Reid JM, Ames MM, Hardwick JS, Reilly JF, Loboda A, Nebozhyn M, Fantin VR, Richon VM, Scheithauer B, Giannini C, Flynn PJ, Moore DF, Zwiebel J (2009). Phase II trial of vorinostat in recurrent glioblastoma multiforme: a north central cancer treatment group study. J Clin Oncol.

[12] Galanis E, Anderson SK, Lafky JM, Uhm JH, Giannini C, Kumar SK, Kimlinger TK, Northfelt DW, Flynn PJ, Jaeckle KA, Kaufmann TJ, Buckner JC (2013). Phase II study of bevacizumab in combination with sorafenib in recurrent glioblastoma (N0776): a north central cancer treatment group trial. Clin Cancer Res.

[13] Krauze AV, Myrehaug SD, Chang MG, Holdford DJ, Smith S, Shih J, Tofilon PJ, Fine HA, Camphausen K (2015). A phase 2 study of concurrent radiation therapy, temozolomide, and the histone deacetylase inhibitor valproic acid for patients with glioblastoma. Int J Radiat Oncol Biol Phys.

[14] Derissen EJ, Beijnen JH, Schellens JH (2013). Concise drug review: azacitidine and decitabine. Oncologist.

[15] Kelly TK, De Carvalho DD, Jones PA (2010). Epigenetic modifications as therapeutic targets. Nature biotechnology.

[16] Aldape K, Zadeh G, Mansouri S, Reifenberger G, von Deimling A (2015). Glioblastoma: pathology, molecular mechanisms and markers. Acta Neuropathol.

[17] Ohgaki H, Kleihues P (2013). The definition of primary and secondary glioblastoma. Clin Cancer Res.

[18] Lamborn KR, Chang SM, Prados MD (2004). Prognostic factors for survival of patients with glioblastoma: recursive partitioning analysis. Neuro Oncol.

[19] Shabason JE, Tofilon PJ, Camphausen K (2011). Grand rounds at the National Institutes of Health: HDAC inhibitors as radiation modifiers, from bench to clinic. J Cell Mol Med.

[20] Ng K, Kim R, Kesari S, Carter B, Chen CC (2012). Genomic profiling of glioblastoma: convergence of fundamental biologic tenets and novel insights. J Neurooncol.

[21] Wen PY, Kesari S (2008). Malignant gliomas in adults. N Engl J Med.

[22] Verhaak RG, Hoadley KA, Purdom E, Wang V, Qi Y, Wilkerson MD, Miller CR, Ding L, Golub T, Mesirov JP, Alexe G, Lawrence M, O’Kelly M, Tamayo P, Weir BA, Gabriel S (2010). Integrated genomic analysis identifies clinically relevant subtypes of glioblastoma characterized by abnormalities in PDGFRA, IDH1, EGFR, and NF1. Cancer cell.

[23] Walker C, DG du Plessis, Joyce KA, Fildes D, Gee A, Haylock B, Husband D, Smith T, Broome J, Warnke PC (2005). Molecular pathology and clinical characteristics of oligodendroglial neoplasms. Ann Neurol.

[24] Zorzan M, Giordan E, Redaelli M, Caretta A, Mucignat-Caretta C (2015). Molecular targets in glioblastoma. Future Oncology.

[25] Noushmehr H, Weisenberger DJ, Diefes K, Phillips HS, Pujara K, Berman BP, Pan F, Pelloski CE, Sulman EP, Bhat KP, Verhaak RG, Hoadley KA, Hayes DN, Perou CM, Schmidt HK, Ding L (2010). Identification of a CpG island methylator phenotype that defines a distinct subgroup of glioma. Cancer cell.

[26] Phillips HS, Kharbanda S, Chen R, Forrest WF, Soriano RH, Wu TD, Misra A, Nigro JM, Colman H, Soroceanu L, Williams PM, Modrusan Z, Feuerstein BG, Aldape K (2006). Molecular subclasses of high-grade glioma predict prognosis, delineate a pattern of disease progression, and resemble stages in neurogenesis. Cancer cell.

[27] Kanu OO, Mehta A, N Di C Lin, Bortoff K, Bigner DD, Yan H, Adamson DC (2009). Glioblastoma multiforme: a review of therapeutic targets. Expert Opin Ther Targets.

[28] Walker MD, Alexander E, Hunt WE, MacCarty CS, Mahaley MS, Mealey J, Norrell HA, Owens G, Ransohoff J, Wilson CB, Gehan EA, Strike TA (1978). Evaluation of BCNU and/or radiotherapy in the treatment of anaplastic gliomas. A cooperative clinical trial. J Neurosurg.

[29] Coffey RJ, Lunsford LD, Taylor FH (1988). Survival after stereotactic biopsy of malignant gliomas. Neurosurgery.

[30] Nishikawa R (2010). Standard therapy for glioblastoma—a review of where we are. Neurol Med Chir (Tokyo).

[31] Hegi ME, Diserens AC, Gorlia T, Hamou MF, de Tribolet N, Weller M, Kros JM, Hainfellner JA, Mason W, Mariani L, Bromberg JE, Hau P, Mirimanoff RO, Cairncross JG, Janzer RC, Stupp R (2005). MGMT gene silencing and benefit from temozolomide in glioblastoma. N Engl J Med.

[32] Ferrara N, Hillan KJ, Novotny W (2005). Bevacizumab (Avastin), a humanized anti-VEGF monoclonal antibody for cancer therapy. Biochem Biophys Res Commun.

[33] Lau D, Magill ST, Aghi MK (2014). Molecularly targeted therapies for recurrent glioblastoma: current and future targets. Neurosurg Focus.

[34] Reardon DA, Desjardins A, Peters KB, Gururangan S, Sampson JH, McLendon RE, Herndon JE, A 2nd Bulusu, Threatt S, Friedman AH, Vredenburgh JJ, Friedman HS (2012). Phase II study of carboplatin, irinotecan, and bevacizumab for bevacizumab naive, recurrent glioblastoma. J Neurooncol.

[35] Reardon DA, Herndon JE, KB 2nd Peters, Desjardins A, Coan A, Lou E, Sumrall AL, Turner S, Lipp ES, Sathornsumetee S, Rich JN, Sampson JH, Friedman AH (2012). Bevacizumab continuation beyond initial bevacizumab progression among recurrent glioblastoma patients. Br J Cancer.

[36] Vredenburgh JJ, Desjardins A, Kirkpatrick JP, Reardon DA, Peters KB, Herndon JE, J 2nd Marcello, Bailey L, Threatt S, Sampson J, Friedman A, Friedman HS (2012). Addition of bevacizumab to standard radiation therapy and daily temozolomide is associated with minimal toxicity in newly diagnosed glioblastoma multiforme. Int J Radiat Oncol Biol Phys.

[37] Li Y, Shin D, Kwon SH (2013). Histone deacetylase 6 plays a role as a distinct regulator of diverse cellular processes. Febs J.

[38] Barneda-Zahonero B, Parra M (2012). Histone deacetylases and cancer. Mol Oncol.

[39] Weichert W (2009). HDAC expression and clinical prognosis in human malignancies. Cancer Lett.

[40] Dokmanovic M, Clarke C, Marks PA (2007). Histone deacetylase inhibitors: overview and perspectives. Molecular Cancer Research.

[41] Stunkel W, Campbell RM (2011). Sirtuin 1 (SIRT1): the misunderstood HDAC. J Biomol Screen.

[42] West AC, Johnstone RW (2014). New and emerging HDAC inhibitors for cancer treatment. J Clin Invest.

[43] Staberg M, Michaelsen SR, Rasmussen RD, Villingshoj M, Poulsen HS, Hamerlik P (2016). Inhibition of histone deacetylases sensitizes glioblastoma cells to lomustine. Cell Oncol (Dordr).

[44] Yang R, Wu Y, Wang M, Sun Z, Zou J, Zhang Y, Cui H (2015). HDAC9 promotes glioblastoma growth via TAZ-mediated EGFR pathway activation. Oncotarget.

[45] Imaoka N, Hiratsuka M, Osaki M, Kamitani H, Kambe A, Fukuoka J, Kurimoto M, Nagai S, Okada F, Watanabe T, Ohama E, Kato S, Oshimura M (2012). Prognostic significance of sirtuin 2 protein nuclear localization in glioma: an immunohistochemical study. Oncol Rep.

[46] Lages E, Guttin A, El Atifi M, Ramus C, Ipas H, Dupre I, Rolland D, Salon C, Godfraind C, deFraipont F, Dhobb M, Pelletier L, Wion D, Gay E, Berger F, Issartel JP (2011). MicroRNA and target protein patterns reveal physiopathological features of glioma subtypes. PLoS One.

[47] Romeo SG, Conti A, Polito F, Tomasello C, Barresi V, D La Torre, Cucinotta M, Angileri FF, Bartolotta M, Di Giorgio RM, Aguennouz M (2016). miRNA regulation of Sirtuin-1 expression in human astrocytoma. Oncol Lett.

[48] Feng J, Yan PF, Zhao HY, Zhang FC, Zhao WH, Feng M (2016). SIRT6 suppresses glioma cell growth via induction of apoptosis, inhibition of oxidative stress and suppression of JAK2/STAT3 signaling pathway activation. Oncol Rep.

[49] Deng CX (2009). SIRT1, is it a tumor promoter or tumor suppressor?. Int J Biol Sci.

[50] Pruitt K, Zinn RL, Ohm JE, McGarvey KM, Kang SH, Watkins DN, Herman JG, Baylin SB (2006). Inhibition of SIRT1 reactivates silenced cancer genes without loss of promoter DNA hypermethylation. PLoS Genet.

[51] Marks PA, Xu WS (2009). Histone deacetylase inhibitors: Potential in cancer therapy. J Cell Biochem.

[52] Camphausen K, Scott T, Sproull M, Tofilon PJ (2004). Enhancement of xenograft tumor radiosensitivity by the histone deacetylase inhibitor MS-275 and correlation with histone hyperacetylation. Clin Cancer Res.

[53] Camphausen K, Burgan W, Cerra M, Oswald KA, Trepel JB, Lee MJ, Tofilon PJ (2004). Enhanced radiation-induced cell killing and prolongation of gammaH2AX foci expression by the histone deacetylase inhibitor MS-275. Cancer Res.

[54] Diss E, Nalabothula N, Nguyen D, Chang E, Kwok Y, Carrier F (2014). Vorinostat SAHA promotes hyper-radiosensitivity in wild type p53 human glioblastoma cells. J Clin Oncol Res.

[55] Kim JH, Shin JH, Kim IH (2004). Susceptibility and radiosensitization of human glioblastoma cells to trichostatin A, a histone deacetylase inhibitor. Int J Radiat Oncol Biol Phys.

[56] Baschnagel A, Russo A, Burgan WE, Carter D, Beam K, Palmieri D, Steeg PS, Tofilon P, Camphausen K (2009). Vorinostat enhances the radiosensitivity of a breast cancer brain metastatic cell line grown in vitro and as intracranial xenografts. Mol Cancer Ther.

[57] Folkvord S, Ree AH, Furre T, Halvorsen T, Flatmark K (2009). Radiosensitization by SAHA in experimental colorectal carcinoma models-in vivo effects and relevance of histone acetylation status. Int J Radiat Oncol Biol Phys.

[58] Zhang Y, Jung M, Dritschilo A, Jung M (2004). Enhancement of radiation sensitivity of human squamous carcinoma cells by histone deacetylase inhibitors. Radiat Res.

[59] Geng L, Cuneo KC, Fu A, Tu T, Atadja PW, Hallahan DE (2006). Histone deacetylase (HDAC) inhibitor LBH589 increases duration of gamma-H2AX foci and confines HDAC4 to the cytoplasm in irradiated non-small cell lung cancer. Cancer Res.

[60] Munshi A, Kurland JF, Nishikawa T, Tanaka T, Hobbs ML, Tucker SL, Ismail S, Stevens C, Meyn RE (2005). Histone deacetylase inhibitors radiosensitize human melanoma cells by suppressing DNA repair activity. Clin Cancer Res.

[61] Rogakou EP, Pilch DR, Orr AH, Ivanova VS, Bonner WM (1998). DNA double-stranded breaks induce histone H2AX phosphorylation on serine 139. J Biol Chem.

[62] Blattmann C, Oertel S, Ehemann V, Thiemann M, Huber PE, Bischof M, Witt O, Deubzer HE, Kulozik AE, Debus J, Weber KJ (2010). Enhancement of radiation response in osteosarcoma and rhabdomyosarcoma cell lines by histone deacetylase inhibition. Int J Radiat Oncol Biol Phys.

[63] Kao GD, McKenna WG, Guenther MG, Muschel RJ, Lazar MA, Yen TJ (2003). Histone deacetylase 4 interacts with 53BP1 to mediate the DNA damage response. J Cell Biol.

[64] Storch K, Eke I, Borgmann K, Krause M, Richter C, Becker K, Schrock E, Cordes N (2010). Three-dimensional cell growth confers radioresistance by chromatin density modification. Cancer Res.

[65] Barazzuol L, Jeynes JC, Merchant MJ, Wera AC, Barry MA, Kirkby KJ, Suzuki M (2015). Radiosensitization of glioblastoma cells using a histone deacetylase inhibitor (SAHA) comparing carbon ions with X-rays. Int J Radiat Biol.

[66] LM Berghauser Pont, Spoor JK, Venkatesan S, Swagemakers S, Kloezeman JJ, Dirven CM, van der Spek PJ, Lamfers ML, Leenstra S (2014). The Bcl-2 inhibitor Obatoclax overcomes resistance to histone deacetylase inhibitors SAHA and LBH589 as radiosensitizers in patient-derived glioblastoma stem-like cells. Genes Cancer.

[67] Chinnaiyan P, Cerna D, Burgan WE, Beam K, Williams ES, Camphausen K, Tofilon PJ (2008). Postradiation sensitization of the histone deacetylase inhibitor valproic acid. Clin Cancer Res.

[68] Singh MM, Manton CA, Bhat KP, Tsai WW, Aldape K, Barton MC, Chandra J (2011). Inhibition of LSD1 sensitizes glioblastoma cells to histone deacetylase inhibitors. Neuro Oncol.

[69] Xu J, Sampath D, Lang FF, Prabhu S, Rao G, Fuller GN, Liu Y, Puduvalli VK (2011). Vorinostat modulates cell cycle regulatory proteins in glioma cells and human glioma slice cultures. J Neurooncol.

[70] Yin D, Ong JM, Hu J, Desmond JC, Kawamata N, Konda BM, Black KL, Koeffler HP (2007). Suberoylanilide hydroxamic acid, a histone deacetylase inhibitor: effects on gene expression and growth of glioma cells in vitro and in vivo. Clin Cancer Res.

[71] Lee MG, Wynder C, Bochar DA, Hakimi MA, Cooch N, Shiekhattar R (2006). Functional interplay between histone demethylase and deacetylase enzymes. Mol Cell Biol.

[72] Bartkova J, Hamerlik P, Stockhausen MT, Ehrmann J, Hlobilkova A, Laursen H, Kalita O, Kolar Z, Poulsen HS, Broholm H, Lukas J, Bartek J (2010). Replication stress and oxidative damage contribute to aberrant constitutive activation of DNA damage signalling in human gliomas. Oncogene.

[73] Squatrito M, Brennan CW, Helmy K, Huse JT, Petrini JH, Holland EC (2010). Loss of ATM/Chk2/p53 pathway components accelerates tumor development and contributes to radiation resistance in gliomas. Cancer cell.

[74] Venere M, Hamerlik P, Wu Q, Rasmussen RD, Song LA, Vasanji A, Tenley N, Flavahan WA, Hjelmeland AB, Bartek J, Rich JN (2014). Therapeutic targeting of constitutive PARP activation compromises stem cell phenotype and survival of glioblastoma-initiating cells. Cell Death Differ.

[75] Dungey FA, Caldecott KW, Chalmers AJ (2009). Enhanced radiosensitization of human glioma cells by combining inhibition of poly(ADP-ribose) polymerase with inhibition of heat shock protein 90. Mol Cancer Ther.

[76] Aldana-Masangkay GI, Sakamoto KM (2011). The role of HDAC6 in cancer. J Biomed Biotechnol.

[77] Inoue A, Yoshida N, Omoto Y, Oguchi S, Yamori T, Kiyama R, Hayashi S (2002). Development of cDNA microarray for expression profiling of estrogen-responsive genes. J Mol Endocrinol.

[78] Sakuma T, Uzawa K, Onda T, Shiiba M, Yokoe H, Shibahara T, Tanzawa H (2006). Aberrant expression of histone deacetylase 6 in oral squamous cell carcinoma. Int J Oncol.

[79] Bazzaro M, Lin Z, Santillan A, Lee MK, Wang MC, Chan KC, Bristow RE, Mazitschek R, Bradner J, Roden RB (2008). Ubiquitin proteasome system stress underlies synergistic killing of ovarian cancer cells by bortezomib and a novel HDAC6 inhibitor. Clin Cancer Res.

[80] Wang Z, Hu P, Tang F, Lian H, Chen X, Zhang Y, He X, Liu W, Xie C (2016). HDAC6 promotes cell proliferation and confers resistance to temozolomide in glioblastoma. Cancer Lett.

[81] Dasmahapatra G, Patel H, Friedberg J, Quayle SN, Jones SS, Grant S (2014). *In vitro* and *in vivo* interactions between the HDAC6 inhibitor ricolinostat (ACY1215) and the irreversible proteasome inhibitor carfilzomib in non-Hodgkin lymphoma cells. Mol Cancer Ther.

[82] Santo L, Hideshima T, Kung AL, Tseng JC, Tamang D, Yang M, Jarpe M, van Duzer JH, Mazitschek R, Ogier WC, Cirstea D, Rodig S, Eda H (2012). Preclinical activity, pharmacodynamic, and pharmacokinetic properties of a selective HDAC6 inhibitor, ACY-1215, in combination with bortezomib in multiple myeloma. Blood.

[83] Morrison BE, D’Mello SR (2008). Polydactyly in mice lacking HDAC9/HDRP. Exp Biol Med (Maywood).

[84] Tao R, de Zoeten EF, Ozkaynak E, Chen C, Wang L, Porrett PM, Li B, Turka LA, Olson EN, Greene MI, Wells AD, Hancock WW (2007). Deacetylase inhibition promotes the generation and function of regulatory T cells. Nat Med.

[85] Zhang CL, McKinsey TA, Chang S, Antos CL, Hill JA, Olson EN (2002). Class II histone deacetylases act as signal-responsive repressors of cardiac hypertrophy. Cell.

[86] Choi YW, Bae SM, Kim YW, Lee HN, Kim YW, Park TC, Ro DY, Shin JC, Shin SJ, Seo JS, Ahn WS (2007). Gene expression profiles in squamous cell cervical carcinoma using array-based comparative genomic hybridization analysis. Int J Gynecol Cancer.

[87] Milde T, Oehme I, Korshunov A, Kopp-Schneider A, Remke M, Northcott P, Deubzer HE, Lodrini M, Taylor MD, von Deimling A, Pfister S, Witt O (2010). HDAC5 and HDAC9 in medulloblastoma: novel markers for risk stratification and role in tumor cell growth. Clin Cancer Res.

[88] Bradbury CA, Khanim FL, Hayden R, Bunce CM, White DA, Drayson MT, Craddock C, Turner BM (2005). Histone deacetylases in acute myeloid leukaemia show a distinctive pattern of expression that changes selectively in response to deacetylase inhibitors. Leukemia.

[89] Zhang Z, Wang Y, Chen J, Tan Q, Xie C, Li C, Zhan W, Wang M (2016). Silencing of histone deacetylase 2 suppresses malignancy for proliferation, migration, and invasion of glioblastoma cells and enhances temozolomide sensitivity. Cancer Chemother Pharmacol.

[90] Rasheed W, Bishton M, Johnstone RW, Prince HM (2008). Histone deacetylase inhibitors in lymphoma and solid malignancies. Expert Review of Anticancer Therapy.

[91] Rasheed WK, Johnstone RW, Prince HM (2007). Histone deacetylase inhibitors in cancer therapy. Expert Opin Investig Drugs.

[92] Ugur HC, Ramakrishna N, Bello L, Menon LG, Kim SK, Black PM, Carroll RS (2007). Continuous intracranial administration of suberoylanilide hydroxamic acid (SAHA) inhibits tumor growth in an orthotopic glioma model. J Neurooncol.

[93] Jordan JT, Wen PY (2015). Novel chemotherapeutic approaches in adult high-grade gliomas. Cancer Treat Res.

[94] Anne M, Sammartino D, Barginear MF, Budman D (2013). Profile of panobinostat and its potential for treatment in solid tumors: an update. Onco Targets Ther.

[95] Lee EQ, Reardon DA, Schiff D, Drappatz J, Muzikansky A, Grimm SA, Norden AD, Nayak L, Beroukhim R, Rinne ML, Chi AS, Batchelor TT, Hempfling K (2015). Phase II study of panobinostat in combination with bevacizumab for recurrent glioblastoma and anaplastic glioma. Neuro Oncol.

[96] Chateauvieux S, Morceau F, Dicato M, Diederich M (2010). Molecular and therapeutic potential and toxicity of valproic acid. J Biomed Biotechnol.

[97] Bialer M, Yagen B (2007). Valproic Acid: second generation. Neurotherapeutics.

[98] Camphausen K, Cerna D, Scott T, Sproull M, Burgan WE, Cerra MA, Fine H, Tofilon PJ (2005). Enhancement of in vitro and in vivo tumor cell radiosensitivity by valproic acid. Int J Cancer.

[99] Hosein AN, Lim YC, Day B, Stringer B, Rose S, Head R, Cosgrove L, Sminia P, Fay M, Martin JH (2015). The effect of valproic acid in combination with irradiation and temozolomide on primary human glioblastoma cells. J Neurooncol.

[100] Shao CJ, Wu MW, Chen FR, Li C, Xia YF, Chen ZP (2012). Histone deacetylase inhibitor, 2-propylpentanoic acid, increases the chemosensitivity and radiosensitivity of human glioma cell lines in vitro. Chin Med J (Engl).

[101] Van Nifterik KA, Van den Berg J, Slotman BJ, Lafleur MV, Sminia P, Stalpers LJ (2012). Valproic acid sensitizes human glioma cells for temozolomide and gamma-radiation. J Neurooncol.

[102] Thotala D, Karvas RM, Engelbach JA, Garbow JR, Hallahan AN, DeWees TA, Laszlo A, Hallahan DE (2015). Valproic acid enhances the efficacy of radiation therapy by protecting normal hippocampal neurons and sensitizing malignant glioblastoma cells. Oncotarget.

[103] Zhou Y, Niu J, Li S, Hou H, Xu Y, Zhang W, Jiang Y (2015). Radioprotective effects of valproic acid, a histone deacetylase inhibitor, in the rat brain. Biomed Rep.

[104] Barker CA, Bishop AJ, Chang M, Beal K, Chan TA (2013). Valproic acid use during radiation therapy for glioblastoma associated with improved survival. Int J Radiat Oncol Biol Phys.

[105] Berendsen S, Varkila M, Kroonen J, Seute T, Snijders TJ, Kauw F, Spliet WG, Willems M, Poulet C, Broekman ML, Bours V, Robe PA (2016). Prognostic relevance of epilepsy at presentation in glioblastoma patients. Neuro Oncol.

[106] Weller M, Gorlia T, Cairncross JG, van den Bent MJ, Mason W, Belanger K, Brandes AA, Bogdahn U, Macdonald DR, Forsyth P, Rossetti AO, Lacombe D, Mirimanoff RO (2011). Prolonged survival with valproic acid use in the EORTC/NCIC temozolomide trial for glioblastoma. Neurology.

[107] Ochiai S, Nomoto Y, Yamashita Y, Watanabe Y, Toyomasu Y, Kawamura T, Takada A, Ii N, Kobayashi S, Sakuma H (2016). Roles of Valproic Acid in Improving Radiation Therapy for Glioblastoma: a Review of Literature Focusing on Clinical Evidence. Asian Pacific Journal of Cancer Prevention.

[108] Furumai R, Matsuyama A, Kobashi N, Lee KH, Nishiyama M, Nakajima H, Tanaka A, Komatsu Y, Nishino N, Yoshida M, Horinouchi S (2002). FK228 (depsipeptide) as a natural prodrug that inhibits class I histone deacetylases. Cancer Res.

[109] Whittaker SJ, Demierre MF, Kim EJ, Rook AH, Lerner A, Duvic M, Scarisbrick J, Reddy S, Robak T, Becker JC, Samtsov A, McCulloch W, Kim YH (2010). Final results from a multicenter, international, pivotal study of romidepsin in refractory cutaneous T-cell lymphoma. J Clin Oncol.

[110] Sawa H, Murakami H, Kumagai M, Nakasato M, Yamauchi S, Matsuyama N, Tamura Y, Satone A, Ide W, Hashimoto I, Kamada H (2004). Histone deacetylase inhibitor, FK228, induces apoptosis and suppresses cell proliferation of human glioblastoma cells in vitro and in vivo. Acta Neuropathol.

[111] Iwamoto FM, Lamborn KR, Kuhn JG, Wen PY, Yung WK, Gilbert MR, Chang SM, Lieberman FS, Prados MD, Fine HA (2011). A phase I/II trial of the histone deacetylase inhibitor romidepsin for adults with recurrent malignant glioma: North American Brain Tumor Consortium Study 03-03. Neuro Oncol.

